# Nitric oxide production in the exhaled air of patients with pulmonary tuberculosis in relation to HIV co-infection

**DOI:** 10.1186/1471-2334-8-146

**Published:** 2008-10-24

**Authors:** Jonna Idh, Anna Westman, Daniel Elias, Feleke Moges, Assefa Getachew, Aschalew Gelaw, Tommy Sundqvist, Tony Forslund, Addis Alemu, Belete Ayele, Ermias Diro, Endalkachew Melese, Yared Wondmikun, Sven Britton, Olle Stendahl, Thomas Schön

**Affiliations:** 1 Department of Medical Microbiology, Faculty of Health Sciences, Linköping University, 581 85 Linköping, Sweden; 2 Department of Clinical Microbiology, Kalmar County Hospital, 391 85 Kalmar, Sweden; 3 Armauer Hansen Research Institute, P.O. Box 1005, Addis Ababa, Ethiopia; 4 Microbiology and Tumour Biology Centre, Karolinska Institute, 171 77 Stockholm, Sweden; 5 Department of Clinical Microbiology, Karolinska Hospital, 171 76 Stockholm, Sweden; 6 Gondar College of Medical and Health Sciences, Gondar University, P.O. Box 196, Gondar, Ethiopia; 7 Department of Infectious Diseases, Karolinska Hospital, 171 76 Stockholm, Sweden

## Abstract

**Background:**

Nitric oxide (NO) is essential for host defense in rodents, but the role of NO during tuberculosis (TB) in man remains controversial. However, earlier observations that arginine supplementation facilitates anti-TB treatment, supports the hypothesis that NO is important in the host defense against TB. Local production of NO measured in fractional exhaled air (FeNO) in TB patients with and without HIV co-infection has not been reported previously. Thus, our aim was to investigate levels of FeNO in relation to clinical symptoms and urinary NO metabolites (uNO).

**Methods:**

In a cross sectional study, FeNO and uNO were measured and clinical symptoms, chest x-ray, together with serum levels of arginine, tumor necrosis factor alpha (TNF-alpha) and interleukin 12 (IL-12) were evaluated in sputum smear positive TB patients (HIV+/TB, n = 36, HIV-/TB, n = 59), their household contacts (n = 17) and blood donors (n = 46) from Gondar University Hospital, Ethiopia.

**Results:**

The proportion of HIV-/TB patients with an increased FeNO level (> 25 ppb) was significantly higher as compared to HIV+/TB patients, but HIV+/TB patients had significantly higher uNO than HIV-/TB patients. HIV+ and HIV-/TB patients both had lower levels of FeNO compared to blood donors and household contacts. The highest levels of both uNO and FeNO were found in household contacts. Less advanced findings on chest x-ray, as well as higher sedimentation rate were observed in HIV+/TB patients as compared to HIV-/TB patients. However, no significant correlation was found between FeNO and uNO, chest x-ray grading, clinical symptoms, TNF-alpha, IL-12, arginine levels or sedimentation rate.

**Conclusion:**

In both HIV negative and HIV co infected TB patients, low levels of exhaled NO compared to blood donors and household were observed. Future studies are needed to confirm whether low levels of exhaled NO could be a risk factor in acquiring TB and the relative importance of NO in human TB.

## Background

One third of the world's population is currently infected with *Mycobacterium tuberculosis *(Mtb) and about 2 million people die every year from tuberculosis (TB) [1]. Yet only 5–10% of immunocompetent individuals who are infected by Mtb develop the disease during their lifetime, indicating the presence of effective host immunity [2]. In Africa, HIV is the single most important factor determining the increased incidence of TB in the past years, underlining the synergy between the progress of HIV and TB [3]. The higher risk of HIV patients developing TB could be related to the fact that macrophages that are not activated by CD4+ T cells are unable to restrict the growth of Mtb [4].

Although controversial in humans, nitric oxide (NO) produced by activated macrophages has anti-mycobacterial effects in mice [5]. The increased production of NO from the amino acid arginine in inflammatory cells like macrophages is catalyzed by the inducible nitric oxide synthase [6] and our group has previously shown that supplementation of arginine during anti-TB chemotherapy improves clinical outcome in TB patients without HIV co-infection (HIV-/TB) [7]. NO has a half-life of a few seconds and is converted to nitrite (NO_2_^-^) and nitrate (NO_3_^-^), two stable end products of NO metabolism that can be measured in urine (uNO) [8]. Moreover endogenous NO, produced in the lower and upper respiratory tract, can be measured in exhaled air as fractional exhaled NO (FeNO) using a chemiluminescence NO analyzer [9]. Asthma [10] and viral respiratory tract infection [11] are associated with high FeNO while low levels have been described in cystic fibrosis [12] and HIV-infection [13]. It has been observed that HIV-/TB patients have an increase in FeNO [14], as well as elevated levels of uNO [15].

Our aim was to investigate the levels of FeNO in relation to uNO in sputum smear positive tuberculosis patients (both HIV+ and HIV-), their household contacts and healthy blood donors in Gondar, Ethiopia, in order to investigate local and general production of NO in pulmonary tuberculosis in relation to clinical symptoms and HIV co-infection.

## Methods

### Study subjects and design

Patients with newly diagnosed smear positive tuberculosis (n = 111) were recruited at the Direct Observed Treatment Short-Course (DOTS) Clinic at Gondar University Hospital, Ethiopia. The inclusion criteria were age 15–60 years and AFB (acid fast bacilli) sputum smear positive tuberculosis. The exclusion criteria were hospitalization, smoking, pregnancy, or concomitant disease other than HIV.

In the group of household contacts to smear positive TB patients (HC, n = 21) FeNO was measured, morning urine was collected and a chest x-ray performed. To be included the HC should be part of the household and continuously spend more than 12 h per day together with the TB patient as well as have a chest x-ray without evidence of active tuberculosis or other infectious disease. Exclusion criteria were acute or chronic disease, smoking and antibiotic or corticosteroid treatment.

A community control group of blood donors (BD, n = 63) were asked to bring a morning urine sample and FeNO was measured. Exclusion criteria were antibiotic or corticosteroid treatment, cough, smoking or household contact treated for TB. All patients and blood donors were offered pre and post counseling at the VCT (voluntary counseling and testing) clinic prior to HIV testing according to the hospital routine.

All patients and study subjects were included only after informed consent. The study was approved by Gondar College of Medical and Health Sciences, Gondar University, Ethiopia and by the Regional Ethics Review Board, Linköping, Sweden.

### Analysis of urine

Nitrate (NO_3_^-^) and nitrite (NO_2_^-^) were measured in urine according to the method described by Verdon et al [16] where nitrate was reduced to nitrite by nitrate reductase and measured using the Griess reaction. Nicotinamide adenine dinucleotide phosphate (10 μM, Roche, Bromma, Sweden) was added directly followed by nitrate reductase (10 U/ml, Roche), glucose-6-phosphate (50 mM, Sigma Chemical CO, St Louis, USA) and glucose-6-phosphate dehydrogenase (40 U/ml, Sigma) diluted in phosphate-buffered saline and incubated at room temperature for 45 min. Sulphanilic acid (1%) diluted in phosphatic acid (5%) and N-(1-naphtyl) ethylenediamine (0.1%, Roche) was added and incubated for 10 min before analyzed in triplicates in an ELISA multi well reader at 540 nm (Anthos labtech instrument 2001, Austria).

### Fractional exhaled NO

In all TB patients, FeNO was measured before TB treatment was initiated. The study subjects were asked to inhale a maximum amount of air outside the valve and exhale into the valve connected to the chemiluminescence NO analyzer (NIOX, Aerocrine AB, Sweden), approved by the FDA [9]. The exhalation was visualized on a computer screen to keep the flow rate between 45–55 ml/s and the measurement length to 10 s [9]. Dead space-time was set to 0.5 s. The average of three measurements with the plateau concentration within 2.5 ppb (parts per billion) or 10% was recorded.

### HIV-status

The HIV status of the TB patients was analyzed with Enzygnost Anti-HIV 1/2 Plus (DADE BEHRING, Germany) and confirmed with Vironistika HIV Uni-Form II ag/ab (Biomérieux, France) by using an ELISA multi well reader (Anthos labtech instrument 2001, Austria). The blood donors were analyzed according to the hospital routines with Vironistika HIV Uni-Form II ag/ab Microelisa system.

### Plasma levels of L-arginine

All plasma samples were filtered by centrifugation at 12,000 × g for 90 min in a Microcon YM-3 tube with a cut-off of 3 kDa (Amicon Inc., Beverly, USA). Plasma L-arginine was analyzed using a modified version of the protocol described by Carlberg [17]. The high-performance liquid chromatography (HPLC) system consisted of an Optilab 931 pump (Shimadzu, Tokyo, Japan) and an RF-535 Fluorescence HPLC monitor (Shimadzu), equipped with a 5 mm Microsphere C18 column (25064 mm) from Knauer (Berlin, Germany). An excitation/emission wavelength of 338/425 nm was used. A mobile phase comprising 20% acetonitrile (Fisher Scientific, Leicestershire, UK) in 10 mM KH2PO4 was used at a flow rate of 1 mL·min-1. Precolumn derivatisation of samples was performed with an equal volume of o-pthaldialdehyde reagent solution (Sigma). Levels of plasma L-arginine were transposed from a standard curve constructed from known concentrations (Sigma).

### Serum levels of TNF-alpha and IL-12

The serum levels of TNF-alpha and IL-12 were analyzed using commercial ELISA kits (Quantikine HS, R&D Diagnostics, USA) according to the instructions from the manufacturer.

### Chest x-ray grading

Grading of chest x-ray findings of pulmonary tuberculosis was done according to the National Tuberculosis Association of the USA in normal, minimal, moderately advanced and far advanced tuberculosis [18]. For statistical evaluation this grading was translated to a semi quantitative scale from 0 (normal) to 3 (far advanced tuberculosis). The chest x-rays were read by one single radiologist and reading was blinded for HIV status.

### Statistics

Data are presented as median and interquartile range (25–75%). To compare groups the Mann-Whitney test was used and correlations were tested with Pearson's correlation test (r^2^). A p-value of ≤ 0.05 was regarded as statistically significant.

## Results

### Study objects

95 out of 111 eligible AFB sputum smear positive TB patients were included in the study. The seroprevalence of HIV was 38% (36/95, 14 M (male), 22 F (female), age 29 y (24–34)). 59 patients were HIV negative (28 M, 31 F, age 23 y (20–29)) (Table 1). Among eligible TB patients, 14% (10/69) of HIV- patients and 14% (6/42) of the HIV+ patients were smokers and were excluded.

**Table 1:** Baseline characteristics of study subjects

	**HIV-/TB**	**HIV+/TB**	**HC**	**BD**
	***n = 59***	***n = 36***	***n = 17***	***n = 46***
***Age (years)***	23 (20–29)	29 (24–34)	25 (21–35)	26 (20–37)
***Sex ***				
Female	31	22	12	11
Male	28	14	5	35
***BCG vaccination scar (%)***	7	8	18	7
***Family member treated for TB (%)***	25	36	100	0
***X-ray***	n = 53	n = 33		
Normal (%)	0	3		
Minimal TB (%)	21	36		
Moderately advanced TB (%)	49	49		
Far advanced TB (%)	30	12*		
***Sedimentation rate (mm/h)***	68 (47–80)	83 (70–98)*		
***Body mass index (kg/m***^2^***)***	16.3 (15.5–18.4)	16.4 (14.7–18.1)		
***Estimated weight loss (kg)***	5.0 (3.0–8.0)	6.0 (3.3–9.3)		
***Temperature (°C)***	37.7 (37.2–38.3)	37.9 (37.3–38.5)		
***Fever (%)***	90	100		
***Fever (weeks)***	8.0 (4.0–12.0)	8.4 (4.0–12.0)		
***Cough (weeks)***	10.0 (4.0–16.0)	8.0 (4.0–13.0)		
***Haemoptysis (%)***	27	27		
***Haemoptysis (weeks)***	0.0 (0.0–0.3)	0.0 (0.0–0.2)		

Data are presented as median and inter quartile range. TB (tuberculosis), HIV (human immunodeficiency virus), HC (household contacts), BD (blood donors), BCG (bacillus Calmette-Guérin), SR (sedimentation rate). * p < 0.05 between HIV-/TB and HIV+/TB patients.

17 HC to smear positive TB patients were included (5 M, 12 F, age 25 y (21–35)). The excluded HC (n = 4) consisted of one subject who did not return for the FeNO and uNO measurements and three subjects with pathological chest x-ray findings: one with TB, one with pneumonia and one with a nodular infiltrate. Five HC and one BD did not bring a morning urine sample but were still included and analyzed on the group level of FeNO. In the group of BD (n = 63), 17 subjects were excluded: four were HIV positive (6%), three were found to be treated by antibiotics, three had a family member treated for TB, one had asthma, one had cough and five did not return for their morning FeNO measurement or to leave a urine sample. In total, 46 BD were included (35 M, 11 F, age 26 y (20–37)). BCG vaccination coverage based on the presence of a BCG scar was low in general (Table 1).

### Clinical data

The clinical data are presented in Table 1. There were no differences between HIV+/TB and HIV-/TB patients with regards to the duration and presence of clinical symptoms such as cough and haemoptysis. HIV+/TB patients had significantly increased sedimentation rate compared to HIV-/TB patients (83 mm/h (70–98) vs. 68 mm/h (47–80), p < 0.001). The body mass index (BMI) was relatively low both in the HIV-/TB and HIV+/TB patients (16.3 kg/m^2 ^(15.5–18.4) vs. 16.4 kg/m^2 ^(14.7–18.1)) with an estimated weight loss of 5.0 kg (3.0–8.0) vs. 6.0 kg (3.3–9.3). HIV+/TB patients had significantly less advanced findings on chest x-ray than HIV-/TB patients when the x-ray findings were transferred to a semi quantitative scale from 0 (normal) to 3 (far advanced) (p = 0.019) (table 1).

### L-Arginine, TNF-alpha and IL-12 in TB patients

Levels of L-arginine in plasma samples from 55 HIV negative and 31 HIV+/TB patients were determined by HPLC. Serum levels of the pro-inflammatory cytokines TNF-alpha and IL-12 were measured in a subset of the patients. There was no difference in arginine levels between the two groups (61.1 μM (49.3–78.4) vs. 57.7 μM (49.2–76.8)). Regarding IL-12, there was a trend towards increased levels in HIV-/TB patients (2.1 pg/ml (0.8–5.8), n = 23 vs. 0.9 (0.8–10.9), n = 11; p = 0.07). There was no difference in the levels of TNF-alpha between the groups (HIV-/TB; 6.7 pg/ml (5.1–12.0), HIV+/TB; 8.3 (5.3–11.2)).

### Exhaled NO

**Figure 1:**
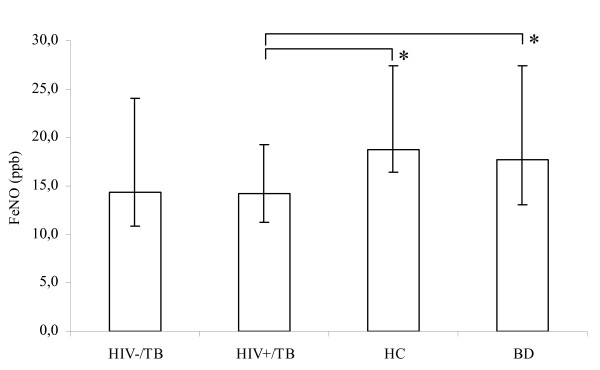
**Levels of exhaled NO (FeNO)**. Levels of exhaled NO (FeNO) in HIV-/TB patients (n = 59), HIV+/TB patients (n = 36), household contacts to smear positive TB patients (HC, n = 17) and blood donors (BD, n = 46) presented as median and interquartile range. ppb (parts per billion). * (p < 0.05).

**Figure 2:**
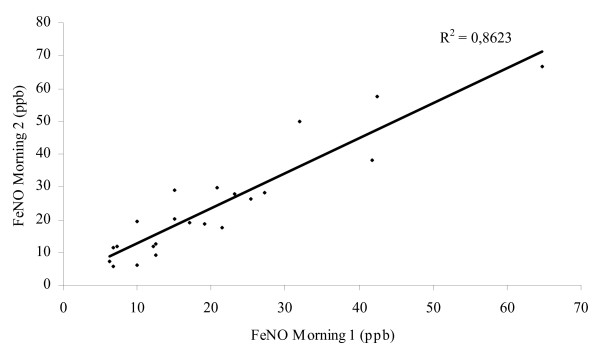
**Consecutive morning measurements of exhaled nitric oxide (FeNO)**. Correlation (r^2 ^= 0.862) between two consecutive morning measurements of exhaled nitric oxide (FeNO) in blood donors and household contacts (n = 22). All measurements were performed between 8 am and 12 am. ppb (parts per billion).

Levels of FeNO in HIV+/TB patients (14.2 ppb (11.3–19.3), n = 36) were significantly lower than in BD (17.7 ppb (13.1–27.4), n = 46, p = 0.013) and HC (18.7 ppb (16.4–27.4), n = 17, p = 0.034) but did not differ from those of HIV-/TB patients (14.3 ppb (10.8–24.0), n = 59) (Figure 1). However, there was a significantly higher proportion of patients with FeNO levels above 25 ppb in the HIV-/TB group compared to HIV+/TB patients (29% (14/49) vs. 8% (2/24), p = 0.05). In the HIV-/TB patients with FeNO levels > 25 ppb there were no differences with regards to sputum smear score, chest x-ray grade or BMI compared to the HIV-/TB patients with FeNO levels < 25 ppb. Although not statistically significant, there was a trend for increased uNO levels in HIV-/TB patients with FeNO > 25 ppb compared to those with FeNO < 25 ppb (1684 vs. 1147 μM, p = 0.140). Measurements of FeNO performed on two consecutive mornings in the blood donors showed a strong correlation (r^2 ^= 0.862, n = 22) (Figure 2). No correlation was found between FeNO and grading of chest x-ray findings, sedimentation rate, levels of arginine, TNF-alpha, IL-12 or clinical symptoms.

### Urine NO^-^_2_/NO^-^_3_

**Figure 3:**
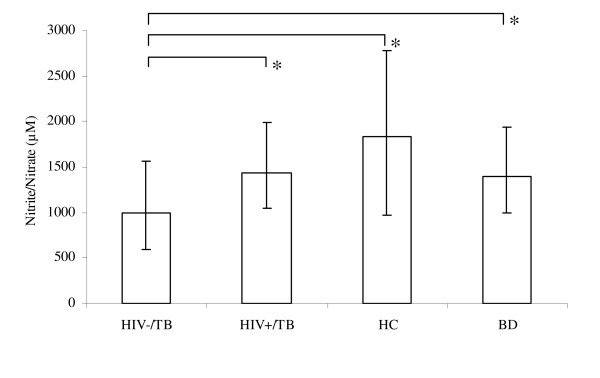
**Levels of urinary nitrite and nitrate (NO_2_^-^/NO_3_^-^)**. Levels of urinary nitrite and nitrate (NO_2_^-^/NO_3_^-^) in HIV-/TB patients (n = 58), HIV+/TB patients (n = 35), household contacts to smear positive TB patients (HC, n = 12) and blood donors (BD, n = 45) presented as median and interquartile range. * (p < 0.05).

Morning urine samples were collected in order to analyze the levels of urinary NO metabolites. HIV+/TB patients were found to have significantly higher levels of urinary NO_2 _^-^/NO_3_^- ^compared to HIV-/TB patients (1431 μM (1044–1991), n = 35, vs. 990 μM (593–1562), n = 58, p = 0.009). HC and BD had higher levels of urinary NO_2_^-^/NO_3_^- ^than HIV-/TB patients (1832 μM (964–2775), n = 12, p = 0.030 and 1394 μM (1000–1939), n = 45, p = 0.034) (Figure 3). No correlation was found between uNO and FeNO in any of the groups.

## Discussion

The level of production and especially the relative importance of nitric oxide in the host defense in human TB remain controversial. There is a lack of clinical studies describing the local production of nitric oxide in smear-positive TB outpatients in high endemic areas with and without HIV co-infection. Here, we have used a new FDA-approved methodology which is routinely used in asthmatic patients [9,10], to measure NO in exhaled air. We found that in HIV positive TB patients there was a lower proportion of subjects with increased FeNO levels (> 25 ppb) and a higher level of urinary nitric oxide metabolites than in HIV negative TB patients. Moreover, in general, patients with sputum smear positive TB had low levels of exhaled nitric oxide compared to control subjects and household contacts.

During active tuberculosis iNOS-mediated generation of NO in alveolar macrophages has been shown [14,19] and elevated levels of FeNO have been recorded in TB patients without HIV co-infection or malnutrition [14]. This is in contrast to the results of the present study which consists of DOTS patients in sub Saharan Africa where most TB patients are treated. HIV- and HIV+/TB patients both had lower levels of FeNO compared to their household contacts and blood donors. Nutritional status could be one explanation for the differences since patients with poor nutritional status were excluded in the previous study measuring FeNO [14].

We found lower levels of arginine in plasma samples from TB patients, both HIV positive and HIV negative, compared to healthy individuals (119 μM) from a previous study in the same area [20]. A recent report showed increased arginase activity in TB patients which could be part of the explanation for a local arginine deficiency in TB [21]. In this study we found no correlation between low levels of arginine and FeNO in TB patients at treatment initiation. However, it has been shown that supplementation of arginine during anti-TB chemotherapy improved clinical outcome and increased arginine levels in HIV-/TB patients [7]. This improvement could be due to an increased NO production, but this hypothesis needs to be confirmed by monitoring NO production during arginine supplementation.

Although both blood donors, household contacts and HIV+/TB patients had significantly increased levels of uNO compared to HIV-/TB patients, our findings of higher uNO levels in HIV+/TB patients compared to HIV-/TB patients are in accordance with a previous study in the same area [15]. It has been shown that HIV+ patients without TB have increased serum nitrate where serum nitrate levels correlated to amount of HIV/DNA in peripheral blood mononuclear cells [22], and that cultured human monocytes infected with HIV-1 expressed iNOS accompanied by a significant production of NO [23]. This might explain the higher levels of urinary NO metabolites in HIV+/TB patients compared to HIV-/TB patients in our population. In this study we found no correlation between serum levels of the pro-inflammatory cytokines IL-12 and TNF-alpha and FeNO or uNO levels in TB regardless of HIV status, although there was a trend for increased IL-12 levels in HIV negative patients.

The control group consisted of blood donors at Gondar University Hospital, Ethiopia, who donate blood to hospitalized relatives. Urinary levels of NO^-^_2_/NO^-^_3 _in this group were higher than in a previous study in Ethiopia (1465 μM (1000–1939), n = 45, vs. mean 1020 ± SD 471 μM, n = 22) [15]. A high exposure to Mtb in the community could be an explanation for the high levels of NO in this group. Measurements of FeNO were performed in the morning and analysis of uNO was done on first morning urine, to minimize the influence of diet [24]. Day-to-day correlation when measuring FeNO at the same time of the day was strong, which allowed us to compare the groups using morning measurements.

There was no correlation between uNO and FeNO in any of the groups indicating a difference in pulmonary NO production and systemic NO production. To ascertain that the levels of FeNO are indicative of the inflammatory status of the lower airways, Wang et al [14] measured NO directly at the level of the vocal cords, main carina, left and right main bronchus and close to the lesion site in the lungs of TB patients. These levels did not differ significantly from the FeNO and correlated highly with FeNO. Other studies have measured exhaled NO at multiple expiratory flows to discriminate between different sources of NO in the lung [25]. This has been used to detect alveolar NO in alveolitis, asthma and chronic obstructive pulmonary disease [25] and indicates that higher expiratory flow rates might be required to measure NO in the terminal airways. We used a flow rate of 45–55 ml/s which is recommended for asthma. As pulmonary TB infection is mainly localized to the lung parenchyma [26], a high expiratory flow rate may be needed to measure NO production optimally in TB patients, but this might be difficult due to the general condition in acute TB.

The normal range of FeNO has been previously described in a healthy European population and the suggested normal range was between 3.6–20.6 pbb in both males and females [27]. Although the levels of FeNO in HIV+/TB patients did not differ from those of HIV-/TB patients there was a significantly higher proportion of patients with FeNO levels above 25 ppb in the HIV-/TB group compared to HIV+/TB patients. Interestingly, we found elevated levels of uNO in the subgroup of HIV-/TB patients with FeNO > 25 ppb and this data indicates that the production of NO in response to TB could be heterogeneous with a subgroup of patients responding with an increased NO production that could be detected both by FeNO and urinary NO metabolites. However, there were no differences in sputum smear score, chest x-ray grade or BMI at treatment initiation with regards to FeNO levels that could support the hypothesis that the levels of FeNO could be associated to the severity of disease. Larger clinical studies including long term clinical follow up will be essential to test this hypothesis thoroughly. We also noted that HIV+/TB patients had significantly less advanced findings on chest x-ray than HIV-/TB patients but no direct correlation between chest x-ray findings and FeNO was observed. The low levels of FeNO in HIV+/TB patients are in agreement with results showing decreased FeNO in HIV+ patients without TB [13]. In HIV-/TB patients we found lower levels of both FeNO and uNO than in blood donors and household contacts. Future prospective follow up studies are needed to test the hypothesis that low NO production in a household contact could be a risk factor for developing TB and whether a high NO production on exposure could be protective against the disease.

## Conclusion

Our study shows locally impaired NO production in the lung but an increased general NO production in HIV co-infected TB patients compared to HIV negative TB patients. Low levels of FeNO compared to blood donors and household contacts were observed in both HIV- and HIV+/TB patients, but future studies are needed to confirm whether low FeNO levels could be a risk factor in acquiring TB, or whether it is a cause of the disease. Monitoring FeNO in TB patients or other vulnerable persons such as household contacts in follow up studies could be a way to identify people at risk of developing TB or to follow up treatment with future NO-based therapeutic strategies.

## Competing interests

The authors declare that they have no competing interests.

## Authors' contributions

TSc, JI, AW, FM and DE carried out the laboratory investigations and field work as well as took part in the design of the project. TSc, DE and JI drafted the manuscript. AA, BA, ED, EM, GA and YW participated in the inclusion and monitoring of patients and the design of the project. GA read the chest x-ray films. AG performed the analysis and interpretation of TNF-alpha and IL-12. TSu and TF took part in the analysis of arginine, the design of the project and the interpretation of data. FM, OS and SB participated in the design and coordination as well as contributed to the drafting of the manuscript. All authors read and approved the final manuscript.

## Pre-publication history

The pre-publication history for this paper can be accessed here:


